# A Story of Three Levels of Sophistication in SCF/KS-DFT Orbital Optimization Procedures

**DOI:** 10.1021/acs.jpca.3c07647

**Published:** 2024-03-14

**Authors:** Daniel Sethio, Emily Azzopardi, Ignacio Fdez. Galván, Roland Lindh

**Affiliations:** † Department of Chemistry—BMC, Uppsala University, P.O. Box 576, SE-75123 Uppsala, Sweden; ‡ Department of Chemistry—Ångström, Uppsala University, P.O. Box 538, SE-75121 Uppsala, Sweden; § Uppsala Center for Computational Chemistry (UC3), Uppsala University, P.O. Box 576, SE-751 23 Uppsala, Sweden

## Abstract

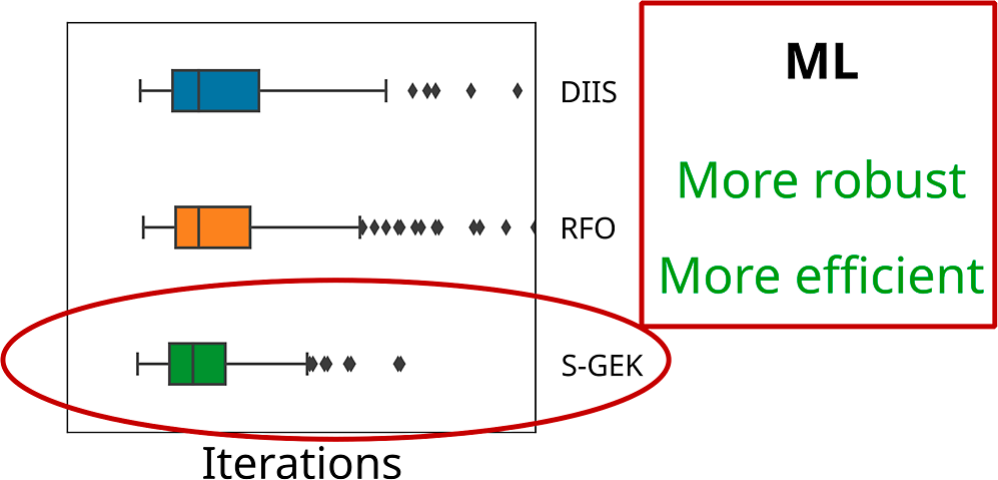

In this work, three versions of self-consistent field/Kohn–Sham density functional theory (SCF/KS-DFT) orbital optimization are described and benchmarked. The methods are a modified version of the geometry version of the direct inversion in the iterative subspace approach (which we call r-GDIIS), the modified restricted step rational function optimization method (RS-RFO), and the novel subspace gradient-enhanced Kriging method combined with restricted variance optimization (S-GEK/RVO). The modifications introduced are aimed at improving the robustness and computational scaling of the procedures. In particular, the subspace approach in S-GEK/RVO allows the application to SCF/KS-DFT optimization of a machine learning technique that has proven to be successful in geometry optimizations. The performance of the three methods is benchmarked for a large number of small- to medium-sized organic molecules, at equilibrium structures and close to a transition state, and a second set of molecules containing closed- and open-shell transition metals. The results indicate the importance of the resetting technique in boosting the performance of the r-GDIIS procedure. Moreover, it is demonstrated that already at the inception of the subspace version of GEK to optimize SCF wave functions, it displays superior and robust convergence properties as compared to those of the standard state-of-the-art SCF/KS-DFT optimization methods.

## Introduction

The optimization of the orbitals in computer implementations of the Hartree–Fock–Roothaan procedure^1^ has been a central issue in computational chemistry for the last 70 years. A similar procedural approach is also at the core of the optimization of the noninteracting orbital in the Kohn–Sham (KS) approach to density functional theory (DFT).^2^ A plethora of reports have been published on suggested improvements, with respect to both efficiency and convergence robustness. Early improvements include a third-order truncated Taylor expansion of the energy parameterized in terms of unitary rotations as suggested by Yaffe and Goddard,^3^ the quadratically convergent self-consistent field (QC-SCF) procedure of Bacskay,^4^ and the use of the direct inversion in the iterative subspace (DIIS) method as designed by Pulay.^5,6^

More recently, a number of variations of the DIIS method have been suggested.^7−14^ The augmented Roothaan–Hall approach is an alternative approach, sometimes used in conjunction with the DIIS approach.^15−19^ A number of restricted-step second-order methods have also been presented and implemented.^20−25^ Alternatively, new hybrid methods have been devised to utilize the pros of second-order methods and the DIIS approach.^26,27^ Overall, these methods generally provide satisfactory convergence statistics. However, as the electronic structure of the target molecule starts to exhibit near-degeneracy effects and open-shell characteristics, it is not uncommon to experience poor convergence or no convergence at all. Further developments of SCF/KS-DFT orbital optimization procedures are needed to address these issues.

What all of the approaches mentioned above have in common is the use of a surrogate model, which is based on a truncated Taylor expansion, usually curtailed after the second-order term. Variations can depend on the parameterization of the energy expression in terms of molecular orbital coefficients, elements of matrices representing unitary rotations of reference orbitals, or one-particle density matrices. Additional contrasts between the suggested schemes are due to ad hoc procedures for trust region and trust radius implementations. These procedures are in general efficient and stable; however, plenty of examples of slow or nonexistent convergence are observed in situations, where alternative solutions to the Fock equations are closely packed in the parameter space around the desired stationary point.

The aim of this paper is to offer improvements of existing optimization techniques and to introduce new tools in the pursuit of an efficient and robust orbital optimization procedure. In the latter case, the techniques from machine learning (ML) will be explored. Rather than fit data (orbital coefficients and associated one-particle density and Fock matrices) to a fixed surrogate model, a flexible surrogate model will be fitted to the data with no loss of information. In particular, the study will investigate the use of a nonparametric regression approach using Gaussian process regression (GPR) for the optimization of SCF and KS-DFT orbitals. The hypothesis is that such an approach is superior to standard and modified quasi-Newton optimization procedures. To explicitly challenge this hypothesis, the novel approach will be benchmarked against two updated standard methods—the GDIIS^28,29^ and the RS-RFO approach, the former with a new resetting approach (r-GDIIS) and the latter as adapted to SCF optimization. The new ML-based optimization procedure will be built on a version of the restricted variance optimization (RVO) procedure^30^ specially adapted to orbital optimizations and the large parameter spaces in such procedures, the subspace version of gradient-enhanced Kriging^31−33^ (S-GEK) approach.

The rest of the paper is structured as follows. The first section will be devoted to the theory and improvements of the GDIIS, RS-RFO, and the new adapted S-GEK approach. Another section follows in which an adequate benchmark suite is designed to effectively test the hypothesis. The results are discussed in the following section, in which the acquired benchmark data are presented and critically analyzed. Finally, the review ends with some conclusions and perspectives. As additional material, an Appendix is included to describe the startup procedure used in the calculations.

## Theory

This section will describe three procedures of SCF/KS-DFT orbital optimization in some detail: the direct inversion in the iterative subspace, the restricted-step rational function optimization (RS-RFO), and the S-GEK in association with restricted-variance optimization (RVO). All of them, however, will have in common the parameterization of the SCF wave function or the determinant describing the KS-DFT noninteracting orbitals, which will be briefly described here before the presentation of the different optimization procedures.

Rather than directly minimizing the SCF/KS-DFT energy with respect to the molecular orbital coefficients, the presented methods will start from a set of orthonormal orbitals, some being occupied and some virtual. The optimization procedure subsequently determines the optimal occupied orbitals via a unitary rotation of the orbitals. It is here noted that the SCF/KS-DFT energy is invariant with unitary rotations of the occupied orbitals among each other. Moreover, the SCF/KS-DFT energy is also invariant to the virtual orbitals; they just span a complementary space to the one spanned by the occupied orbitals. Henceforth, {*i*, *j*, *k*}, {*a*, *b*, *c*}, and {*p*, *q*, *r*}, respectively, will denote occupied, virtual, and general orbital indices. It is noted that the optimization procedure focuses exclusively on the rotations between the occupied and virtual subspaces of the SCF/KS-DFT orbitals. Following the description suggested by Jørgensen and co-workers,^34,35^ this parameterization starts with a reference function, a Slater determinant, |Ψ_0_⟩, defining the number of electrons and the occupied orbitals
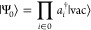
1where *a*_*i*_^†^ (*a*_*i*_) is a creation (annihilation) operator creating orbital *i* and ∏_*i*∈0_*a*_*i*_^†^ is an ordered product of such creation operators acting on the vacuum state, |vac⟩.

A unitary transformation (restricting it to real rotations) of the orbitals making up the SCF/KS-DFT Slater determinant is subsequently described as
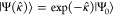
2where
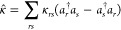
3where **κ** is an antisymmetric matrix and exp(−**κ**) is a unitary matrix.

This renders the SCF/KS-DFT expectation energy to be expressed as
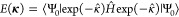
4where *Ĥ* is the Hamiltonian operator. For the purpose of facilitating the orbital optimization, the energy is expressed as a Taylor expansion^27^ (here explicitly expressed up to second order)
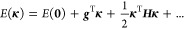
5where the elements of the gradient vector, ***g***, are expressed as
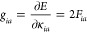
6that is, the matrix elements are, with the exception of a factor of 2, identical to the occupied–virtual elements of the Fock matrix in molecular orbital (MO) basis (not necessarily the canonical orbitals). Finally, for the elements of the Hessian matrix, ***H*** (not to be confused with the Hamiltonian), in a quasi-Newton procedure, only a reasonable approximation is used
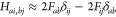
7in association with some Hessian update methods.

For practical purposes, the occupied–occupied and virtual–virtual subblocks of **κ** are set to zero. Hence, the parameter space of the SCF/KS-DFT orbitals is completely defined by the occupied, virtual, or the virtual–occupied subblocks of **κ**. It is noted that the orbitals generated with the parameterization are not necessarily identical to the canonical SCF/KS-DFT orbitals. A postconvergence diagonalization of the occupied–occupied and the virtual–virtual subblocks of the Fock matrix would, however, generate the canonical occupied and virtual orbitals, respectively.

### Direct Inversion in the Iterative Subspace

A number of procedures to optimize the orbitals of a Slater determinant were offered in the early days of the development of the self-consistent field approach of the Hartree–Fock single configuration wave function model. Most of these were either slow to convergence or far from robust. This changed to the better in 1980 when Pulay proposed the use of the so-called direct inversion in the iterative subspace method,^5^ a combination of a variable metric update method and a minimization step as a tractable approach. Originally, this approach was introduced in terms of a parameterization using the occupied–virtual block of the Fock matrix, while here the developments are parallel to those suggested by Fischer and Almlöf.^29^ That is, the parameterization is in terms of the occupied–virtual orbital rotations, as mentioned at the start of this section; the Hessian matrix is not explicitly stored, but it is implicitly updated as it is multiplied by a trial vector with an on-the-fly version of the Broyden–Fletcher–Goldfarb–Shanno (BFGS)^36−39^ method; and the GDIIS^28^ approach is employed. The DIIS method has since its introduction been the subject of many publications and modifications. This report will not dwell into the details of these; however, it is interesting to note that recent publications^40^ indicate that the DIIS method of Pulay actually is a special case of the Anderson mixing-procedure^41^ from 1965.

The GDIIS procedure (using this name to denote the DIIS variant described by Fischer and Almlöf^29^) is a two-step procedure that assumes the presence of a set of *n* parameter vectors, (**κ**_*i*_, *i* = 1, ..., *n*) and the corresponding set of gradient vectors, ***g***_*i*_, one for each iteration, *i*. First, for parameter sets in the quadratic region, an approximated error vector, ***e***_*i*_, can be compiled as
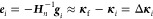
8where ***H***_*n*_ is the updated approximate Hessian and κ_f_ is the optimal parameter set. The *m* last iterations—the iterative subspace—are presumed to be in this quadratic region and are used in the first step of the GDIIS procedure, *m* is the so-called depth of the DIIS procedure. An improved set of parameters can be computed as
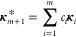
9by minimizing the norm of the extrapolated error vector
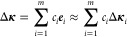
10under the condition that . This is achieved by minimizing the following Lagrangian
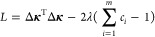
11Here, the first term on the right-hand side (rhs) is expressed as
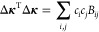
12where *B*_*i*,*j*_ = ***e***_*i*_^T^***e***_*j*_ are the elements of the error matrix. Solving this minimization problem corresponds to finding the solution to the following set of equations
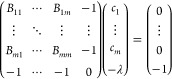
13which is of the type ***B***^*a*^***x*** = ***b***, where ***B***^*a*^ is the augmented error matrix. This is trivially solved as . It is noted that as Δ**κ** converges toward the zero vector, the parameter set for the next iteration will converge toward the optimal parameter set, **κ**_f_. The procedure now goes into the second step in which the convergence is checked by computing a second update to the parameter set, now in the full space of the parameter space, δ**κ**_*n*+1_, as
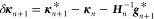
14The actual gradient, ***g***(**κ**_*n*+1_), is computed for the new parameter set. If the norm of the gradient is below the convergence threshold, then the procedure is considered converged. Otherwise, the GDIIS procedure is repeated in a subsequent iteration but now with optionally one more error vector.

### Resetting Direct Inversion in the Iterative Subspace

Experience has demonstrated that solving the system of equations of eq 13 is prone to numerical instability. This issue can be addressed in at least three different ways. First, the linear dependence can be reduced/eliminated by setting *m* to a rather small constant integer value (in the current implementation *m*_max_ = 5). As the GDIIS procedure iterates, error vectors from earlier iterations are eliminated, thus reducing the risk of linear dependence. Second, as suggested by Chupin et al.,^40^ the new error vector is projected onto the manifold of the *m* previous error vectors, and if the norm of the remainder (a measure of the extent of linear dependence) is below a threshold, *m* is either reset to one or incrementally reduced until the condition is no longer fulfilled. This approach is not used in the present benchmarking. Third, a reformulation of the problem is used, as suggested by Sellers,^42^ which reduces the numerical problem—the C^2^-DIIS method. Here, the optimization problem is cast as an eigenvalue problem
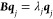
15which implies a normalization of the eigenvectors according to . The selection of the appropriate eigenvector starts by computing the coefficients of the original formulation of the DIIS procedure, from the renormalization ***c***_*j*_ = ***q***_*j*_/*N*_*j*_, where , followed by the evaluation of the norm of the extrapolated error vector. In the original implementation, the set of coefficients corresponding to the lowest eigenvalue above a specific threshold, to avoid solutions that are numerically suspect, is kept. However, in the present benchmark, a modification of this selection procedure is introduced as follows. The norm of ***c***_*j*_ is computed. A large number corresponds to the elements of the coefficient vector being a series of large numbers with varying sign, a signature of a solution corrupted by numerical instability, which is also reflected in a small eigenvalue. Hence, in the present implementation, if ***c***_*j*_^T^***c***_*j*_ > δ and ***c***_*j*_^T^***Bc***_*j*_ < σ, where δ = 100 and σ = 10^–5^, that particular solution is rejected. Otherwise, the solution corresponding to the extrapolated error vector with the lowest norm is selected.

This is not, however, the end of the considerations that need to be attended to for optimal convergence. In what follows is a description of conditions that will reset either the DIIS depth or the BFGS update of the approximate Hessian. This will be coined the resetting DIIS approach, r-DIIS. It should be noted that the suggested procedure below has had its precursors in the restarted DIIS method (r-Pulay)^12^ which on regular intervals resets the depth of the DIIS and the method of Fang and Saad^9^ which resets the DIIS depth if the ratio of the residuals of the two last iterations exceeds a threshold. The suggested approach here, however, contains three conditions that will trigger the resetting mechanism.

First, numerical stability is not the only issue with the DIIS approach, the anharmonic character of the PES is another problem; the norm of the gradient is not linear with respect to the distance from the stationary point. Thus, when the DIIS method is applied to error vectors whose norms differ by orders of magnitude, the predictive power of the approach is lost. In the current implementation, this issue is handled by the condition
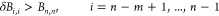
16where δ = 10^–8^. If the condition is fulfilled, the depth of the DIIS procedure, *m*, is decreased in steps of one until the condition is not fulfilled or *m* = 1.

Second, having solved these problems, there could still be convergence issues due to the qualitative nature of the (G)DIIS procedure; in particular, it is a procedure with no reference to the value of the energy. As the method is formulated, it is based on the energy function being convex. That is, this strictly leads to the expectation that the gradient norm continuously decreases as the geometry approaches the stationary point. However, this is not always the case. Especially, it is not uncommon for the optimization procedure to evolve along the energy surface such that it experiences a shoulder (inflection point) in one direction while being at a minimum in all other directions. While propagating on such a shoulder, the gradient norms can be fairly small. However, as the optimization procedure updates the molecular orbitals beyond the edge of the shoulder, a rapid decrease of the energy will initially be associated with a significant increase of the norm of the error vectors. It is clear that the (G)DIIS method in such a case will produce an extrapolated set of coordinates, which returns up to the shoulder, a region of error vectors with a low norm. The present implementation of the GDIIS procedure tries to detect this case by finding the smallest element *B*_*i*,*i*_ and checking the condition *E*_*n*_ + δ < *E*_*i*_, where δ = 10^–4^. Additionally, to ensure the *B*_*jj*_ values are in roughly descending order, it is checked whether any *B*_*ii*_σ < *B*_*j*+1,*j*+1_, with σ = 15. If any of these conditions is fulfilled, the depth of the (G)DIIS procedure, *m*, is reset to 1, and a pure variable metric step is taken based on the gradient of the latest trial.

Third, the updated Hessian is used in the two steps of the GDIIS procedure, and thus, it is important that it be qualitatively correct. Since the error vectors are also a part of these updates, they will only make sense if the gradients are consistent. Thus, under similar conditions as described above, the BFGS procedure might produce updated Hessians that are nonphysical and/or ill-conditioned, usually detected by a series of monotonically smaller and smaller displacements in the GDIIS iterations suddenly interrupted by an unexpectedly large displacement. In the current implementation, this is considered to be the case if the norm of the displacement vector is larger than π—i.e., a rotation larger than 180°. The remedy for this behavior is that a new update vector is computed using a reduced depth of the BFGS-update procedure repeatedly until the norm of the displacement vector is acceptable.

### Restricted-Step Rational-Function Optimization

In the past, the so-called RS-RFO procedure^43,44^ has been applied with success to molecular structure optimizations in association with the use of internal coordinates,^45^ an approximate initial Hessian,^46^ and the BFGS Hessian update method. Inspired by the 1992 paper by Fischer and Almlöf,^29^ in which it was suggested that the successful use of the DIIS method, parameterized over the occupied–virtual block of the Fock matrices,^5,6^ for SCF orbital optimizations could be transferred over to a parameterization of the procedure in terms of orbital rotations, a similar adaptation will be executed here. It is noted that such a transfer is obvious; a new alternative parameterization has emerged, and good estimates of the Hessian matrix exist. The only matter that is a possible restriction in the adaptation of RS-RFO to orbital optimization is the size of the parameter space, which for a given system goes from 3*N* – 6, in the case of a molecular structure optimization (*N* being the number of atoms), to *N*_SCF_ = *N*_occ_ × (*N*_AO_ – *N*_occ_) in the case of a SCF orbital optimization (*N*_AO_ is the total number of linearly independent basis functions and *N*_occ_ is the number of occupied orbitals). That is, for a basis set of reasonable quality in association with second-row atoms, the characteristic dimension of the matrices associated with the RFO procedure increases by 1 order of magnitude with respect to a typical geometry optimization, and the scaling of the procedure might increase by as much as 3 orders of magnitude. Moreover, while 3*N* – 6 scales linearly with the number of atoms, *N*_SCF_ scales quadratically. For a successful implementation, this issue will have to be mitigated.

The basics of the RS-RFO procedure are briefly presented here. It is an alternative to a conventional step-restricted truncated second-order Taylor expansion of the energy in which the energy is expressed by a rational function—a Padé [2/2] approximant—as
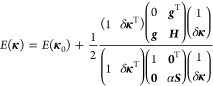
17where δ**κ** = **κ** – **κ**_0_, ***S*** is a matrix usually set to the unit matrix, and α is a parameter which in the case of unconstrained optimizations is set to unity, but in the case of constrained optimizations, it is adjusted to get a displacement with a norm within the step-restriction length.

The stationary points of the RS-RFO equation are found as solutions to eigenvalue equations
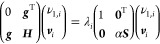
18such that the computed eigenvectors are associated with the displacement vectors as
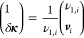
19The critical part of the implementation of the solver to the eigenvalue problem is (i) only the lowest eigenvalue is needed, and (ii) the full Hessian matrix is too large for explicit storage. This is trivially solved as follows. First, the eigenvalue problem will be solved using the iterative method of Davidson,^47^ which will significantly reduce the computational expense and storage requirements as compared to those of any general procedure finding all eigenvalues. Second, in the iterative procedure, the on-the-fly application of the BFGS update, as suggested by Fischer and Almlöf,^29^ was implemented. As in the case of the implementation of the GDIIS method, mentioned above, the depth of the BFGS procedure is modified if the suggested step length is longer than π. In that case, the depth of the BFGS update is set to 1, and the step restriction is set to π (the default value is 10.0 if *m* ≠ 1, such that poor BFGS updates can be diagnosed and proper care can be taken).

To conclude this section, it is appropriate to mention the recent implementation by Slattery et al. of a quasi-Newton unitary optimization with trust-region approach.^48^ There are many similarities to what is proposed above. The main differences are that Slattery et al. used the low-memory version of BFGS, the L-BFGS method,^49^ the original step is computed with a conventional quasi-Newton step, an optional line search, and a subsequent step restriction is implemented by the use of a trust radius. The trust radius is compiled from the most recent line search.

### Gradient-Enhanced Kriging

The GEK^31−33^ approach—a GPR^50^ variant—is an ML method that has been applied with significant success to, for example, molecular structure optimizations.^30,51−53^ In this report, such a GEK implementation especially modified for the case of SCF orbital optimizations is presented. The size of the parameter space, *N*_SCF_, however, is a significant problem for an efficient implementation, as reported by Ritterhoff,^54^ who demonstrated that the GEK procedure applied to SCF orbital optimization can have competitive iteration counts, while the timings are very unfavorable due to the *N*^3^ scaling of the procedure. The author of this report proposes, though, that the general *effective* dimensionality of the SCF optimization problem is much smaller than the formal size. Considering, for example, that the GDIIS procedure normally converges in 15–25 iterations, using a depth of 4–5 iterations, one has to conclude that the effective dimensionality is much smaller than what one expects—this is mainly because the different degrees of freedom are to a large extent uncoupled. Hence, inspired by the “smallness” of the DIIS formulation and the structure of second-rank Hessian-update methods, a subspace formalism of the GEK procedure (S-GEK), adapted for SCF/KS-DFT orbital optimizations, will be put forward. However, before presenting the space-reduction procedure, the basics of the GEK implementation is introduced here. The details of this procedure have been published elsewhere.^30,51,52^

The GEK is a gradient-enhanced GPR; that is, not only the energy but also all elements of the gradient are regressed. Hence, the surrogate model reproduces exactly the energy and the complete gradient vectors at the points of regression; the model is fitted to the data, not the reverse.

The surrogate model is effectively expressed as
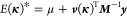
20where μ is the bias/trend function (can be a constant or a function of **κ**), ***v*** is the generalized covariance vector, ***M*** is the generalized covariance matrix, and ***y*** is the generalized value vector. This can also alternatively be expressed as
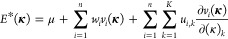
21where *w*_*i*_ and *u*_*i*,*k*_ are weights derived from the expression ***M***^–1^***y***, *n* is the depth of the GEK, that is, the number of sets of coordinates for which the energy and gradient vector is known, and, finally, *K* is the dimensionality of the parameter space.

Although in eq 21 the contributions from *v*_*i*_(**κ**) and ∂*v*_*i*_(**κ**)/∂(κ)_*k*_ are written separately, in practice, they are all collected in a single list, such that each element of the vectors ***v***(**κ**) and ***y*** or the matrix ***M*** refers to either the energy or the derivative at a data point *i* in some predefined order. For instance, one could sort first the energies for all data points and then all derivatives for the first data point and the derivatives for the second data point, etc. Thus, the ***y*** vector would be (expressed as the transpose of a row vector for convenience)
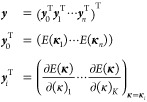
22where **κ**_*i*_ indicates the coordinate vector of data point *i*, while (κ)_*k*_ denotes the *k*th dimension or the *k*th component of the **κ** vector. That is, the ***y*** vector collects all of the energies and gradients from the *n* previous iterations, which the surrogate model will reproduce exactly. The ***M*** matrix collects the covariance between the data points, as well as the first and second derivatives
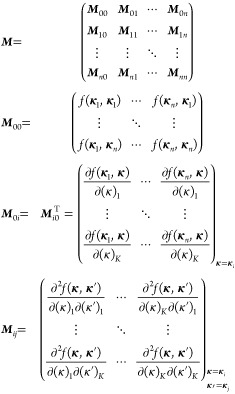
23and the vector ***v***(**κ**) collects the covariance and derivatives between an arbitrary point **κ** and the data points **κ**_*i*_
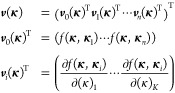
24The functions *v*_*i*_(**κ**) are therefore just a shorthand notation for *f*(**κ**, **κ**_*i*_), and the weights *w*_*i*_ and *u*_*i*,*k*_ in eq 21 are obtained as
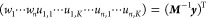
25

A crucial element of the GEK procedure is the definition of the covariance function *f*(**κ**, **κ′**) (a symmetric function), which is the building block of the covariance vector and matrix. The covariance function is a mathematical description of the correlation between the values of two data points. Many choices are possible for a valid covariance function,^55^ and based on the successful experience with geometry optimization,^30,51,53^ we chose a Matérn 5/2 covariance function^56^ with individual characteristic length for each dimension. A metric is defined to measure the distance, in parameter space, between two data points as
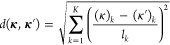
26where *l*_*k*_ is the characteristic length, defined below. Note that this reduces to a simple Euclidean distance if all *l*_*k*_ = 1. The covariance function then reads
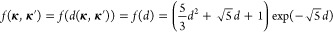
27which has a Gaussian-like shape (in fact, a Gaussian or “squared exponential” would also be a possible covariance function to use). The internal regression parameterization is based on the eigenvectors of the approximate Hessian of the latest iteration, that is, the **κ** vector used in the whole of this section is actually **κ̃** = ***U*κ**, where ***U*** is the matrix of eigenvectors of the approximate Hessian and **κ** is the vector of “raw” occupied–virtual orbital rotation parameters in the current orbital basis.

When the model is built with a single data point, using a Matérn 5/2 covariance function, the Hessian matrix at that point is diagonal with eigenvalues
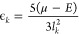
28This observation is used to derive the associated characteristic length for each dimension, in the representation that diagonalizes the Hessian, as
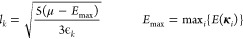
29considering that in this implementation μ – *E*_max_ = 10.0 *E*_h_. This has the effect of reproducing the approximate Hessian when a single data point is used to build the model.

The GEK does not only allow for prediction of energy values but also to each such prediction associate an estimate of the accuracy of that particular value—the expected variance
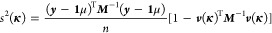
30This facilitates an analytically based robustness measure to be applied to the procedure; rather than to make the optimization procedure restricted by an arbitrary step length, a variance restriction can be applied. This has been expressed in the RVO, which has with success been used in molecular structure optimizations.^30,51,53^

### Subspace Gradient-Enhanced Kriging

To proceed to the novel contribution reported in this work, the parameter space reduction, let us consider some technical details of the DIIS and the quasi-Newton procedure. In the first case, satisfactory convergence rates with DIIS are achieved by projecting the multidimensional error vectors, expressed either as displacement vectors or as gradients, of the last few iterations onto a small subspace spanned by the error vectors. This defines the ***B*** matrix, which is used to solve the DIIS equations. Subsequently, a single quasi-Newton step in the full parameter space is performed on the residual error vector. In the second case, the Hessian update of the quasi-Newton procedure is the fundamental tool toward convergence acceleration. It is noted that, for example, the BFGS second-rank update procedure can technically be described as the following update
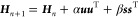
31where α and β are some real constants, and ***u*** = Δ***g*** and ***s*** = ***H***Δ**κ**. In particular, it is noted that it is these vectors that communicate efficiently the most significant parts of the coupling between the parameters. It is the availability of an accurate Hessian approximation, obtained through this procedure, that to a large extent explains the much faster convergence of the quasi-Newton approach compared to that of a plain conjugate gradient method. Inspired by the efficiency of this approach, a set of unit projection vectors, defined in the full space and a reduced space as ***e***_*i*_^f^ and ***e***_*i*_^r^, respectively, to define an effective subspace was designed by selecting from (a) the actual displacement vectors of the last *m* – 1 iterations, and (b) the associated gradient difference vectors. To this list is further added the gradient vector at the latest point and the vector corresponding to the predicted displacement of the RS-RFO procedure at the current structure. This set of 2*m* vectors in the full space is subsequently orthonormalized to generate ***e***_*i*_^f^, while the corresponding vectors in the reduced space are set to , i.e., they are canonical basis vectors in the reduced space. This defines the projection operator
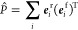
32In what then follows, the original coordinate vectors, gradient vectors, and the approximate Hessian are projected onto this subspace, and the resulting entities are subsequently used in an RVO procedure supported by the GEK approximation. For example, a gradient in the full space, ***g***^f^, is expressed as
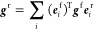
33in the reduced space. The resulting displacement, δ**κ**^r^, from solving the optimization in the reduced space is finally expanded up into the full space by
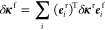
34

This procedure, the S-GEK/RVO or S-RVO approach, can be used as a convergence acceleration approach on top of the previously proposed RS-RFO method. It significantly reduces the dimensionality of the RVO procedure from *m* × (1 + *N*_SCF_) to *m* × (1 + 2*m*), making it a viable option for SCF/KS-DFT orbital optimization.

## Computational Details

The object of the benchmark suite to be designed is to offer a vigorous test of the merits of the three optimization procedures presented in this study. The benchmarking will explore both SCF and DFT optimizations, this in order to explore performance as a function of the different complexities of the nonlinear parameterization of the energy as expressed in SCF vs DFT. Moreover, to evaluate the performance of the three SCF/DFT orbital optimization methods, a comprehensive data set of 2080 target electronic structures was employed, encompassing both organic molecules and transition-metal (TM) complexes. The goal of the data sets was to constitute representative cases of both closed- and open-shell wave functions to contain cases of strained wave functions as represented by nonequilibrium molecular structures and to include cases with potential near-degeneracy effects as represented by TM complexes. In this respect, it is the expectation that the data set presented below is not biased and does not contain tests that are cherry-picked to favor one or another of the optimization methods. Moreover, with this selection of test suite, it comes naturally that any subsequent analysis of the merits of the three methods has to be based on a statistical comparison. This also eliminates the significance of any parameter sensitivity some molecular structures might exhibit in individually compiled convergence rates.

Thus, structures beyond stationary points and structures containing transition metals as well as those beyond the ground electronic states were utilized to evaluate the robustness of the reported SCF/DFT optimization procedures. Among these, a subset of 500 (265 singlets +235 doublets) organic molecules was selected from the Sella^57^ database to represent organic molecules composed of 7–25 atoms. The original Sella database contains molecular structures close to a transition state structure for both singlet and doublet spin states. These structures were subsequently optimized to either the reactant or the product equilibrium molecular structure, thus doubling the number of molecular structures. The two sets of molecular structures in the singlet spin state were subsequently used for the benchmarking of wave functions in both the singlet and triplet states. Hence, the organic molecules were subjected to calculations in singlet, doublet, and triplet states, involving both stationary and transition state structures. Additionally, a subset of 275 structures was drawn from the tmQM^58^ data set to represent TM complexes with fewer than 20 atoms. Here, the TM complexes were computed in both their singlet and triplet electronic states. To summarize: 265 singlet TS-like structures were computed as both singlet and triplet; those structures were optimized to a stable singlet structure, and again, the wave function was computed as singlet and triplet; 235 doublet TS-like structures were computed as doublets; after optimization to stable doublet structures, the doublet wave function was again computed; 275 TM complex structures were computed as singlet and triplet. This gives 265 × 4 + 235 × 2 + 275 × 2 = 2080 total structures for each SCF optimization method. It should be noted that the geometry optimizations were performed only once at the HF/3-21G level of theory, and the resulting structures were used for all reported calculations as single points.

All calculations were carried out using the OpenMolcas software package, version 23.06.^59^ The evaluation of three distinct SCF procedures was conducted by utilizing both HF and DFT-based methods. DFT calculations were done using the B3LYP functional.^60^ For organic molecules, the cc-pVDZ basis set^61−63^ was adopted, while molecules containing TMs were computed using the ANO-R1 basis set^64,65^ [the use of ANO-R1 in OpenMolcas automatically enables the exact 2-component (X2C)^66^ scalar relativistic Hamiltonian and finite-size nuclei]. Singlet calculations were done with the restricted SCF formulation, and doublet and triplet calculations were done with the spin-unrestricted one. The default SCF convergence criteria were utilized (energy change below 1 × 10^–9^*E*_h_, a maximum absolute value of the occupied–virtual Fock matrix below 1.5 × 10^–4^, and a norm of Δ**κ** below 10^–3^), along with the RICD approximation,^67^ while the default LK procedure^68^ was disabled. For DFT calculations, the default Libxc^69^ implementation and numerical quadrature in OpenMolcas were employed. The calculations were conducted without the imposition of any symmetry constraints. The performance of the three SCF procedures was assessed by comparing the number of SCF iterations. If not otherwise stated, this is the iteration count after the standard SCF startup procedure, which consists of both the “initial guess” of occupied orbitals and a number of preliminary iterations with EDIIS^8^ and DIIS^42^ procedures, as implemented in OpenMolcas and described in the Appendix, such that the reported iteration counts refer only to the three methods benchmarked in this work.

## Results and Discussion

The results in this section are presented in two parts. First, the significance of the resetting technique of the r-GDIIS procedure will be assessed as compared to that of a vanilla GDIIS implementation. Second, the different orbital optimization methods, the r-GDIIS, the RS-RFO, and the S-GEK/RVO methods, will be benchmarked against each other.

### Performance Assessment of the r-GDIIS Approach

The suggested resetting technique of the r-GDIIS method is here compared to a straightforward implementation of the GDIIS with respect to iterations until convergence according to the default threshold. The average number of iterations and the standard deviation of the iteration count for the eight separate sets of benchmark calculations as assessed with the HF and the B3LYP methods are presented in Table 1. Additionally, the table contains information, for each set, on the number of molecules for which the calculation took more than 120 iterations to converge and the number of molecules for which convergence was not reached before 400 iterations (including the SCF startup in this latest count). Note that the statistics are based on the cases in which convergence was reached. Hence, an apparent advantage from the statistics could be a sign that problematic cases did not converge and, in this sense, did not contribute to the evaluation of the statistical data.

**Table 1:** Benchmark Results for the Eight Sets of Benchmark Calculations Compiled with the GDIIS and r-GDIIS Approaches^a^

	HF		B3LYP	
	GDIIS	r-GDIIS	GDIIS	r-GDIIS
singlets	11.2(2.5)/0(0)	11.3(2.9)/0(0)	9.4(2.2)/0(3)	9.8(3.1)/0(0)
singlets opt	7.9(1.0)/0(0)	7.9(1.0)/0(0)	8.1(1.4)/0(0)	8.1(1.4)/0(0)
doublets	15.4(5.3)/0(19)	17.1(8.4)/0(0)	11.0(2.4)/0(2)	11.1(3.0)/0(0)
doublets opt	12.9(6.0)/0(11)	15.2(13.4)/0(0)	10.0(3.7)/0(5)	11.4(12.2)/1(0)
triplets	11.5(5.9)/0(4)	12.3(8.1)/0(0)	7.0(2.5)/0(0)	7.0(2.5)/0(0)
triplets opt	12.6(15.8)/1(24)	19.6(32.4)/8(0)	8.6(4.1)/0(0)	8.6(4.1)/0(0)
TM singlets	12.5(4.9)/0(4)	13.2(7.6)/0(0)	10.9(3.6)/0(1)	11.4(3.8)/0(5)
TM triplets	34.0(20.0)/1(102)	47.8(36.9)/7(2)	24.6(29.5)/2(48)	33.5(29.6)/7(2)

a The reported numbers are the average number of iterations post SCF startup (the standard deviation)/the number of molecules for which more than a total of 120 iterations were needed (the number of molecules which did not converge before a total of 400 SCF iterations).

The following three general observations can be noted. First, the B3LYP optimization procedure seems in general to be less problematic to converge compared to HF in the cases of open-shell calculations. Second, the triplet state TM benchmarks stand out as a more challenging task both at the HF and the B3LYP level of theory as compared to the other benchmark sets. Third, convergence is faster for benchmark molecular structures that correspond to equilibrium molecular structures; note that neither triplet benchmark set corresponds to equilibrium molecular structures as they are optimized for a singlet wave function. This is consistent with the purpose of the design of the test suites. Finally, it is clear that the resetting of the DIIS depth results in a significant improvement. For example, the resetting mechanism reduces the total number of failed convergence cases from 164 to 2 and from 59 to 7 for the HF and B3LYP methods, respectively. In particular, it is noted that for the triplet transitions, metals close to half of the test cases fail to converge with the standard GDIIS (102 cases out of 275), whereas the resetting approach more or less eliminates this problem (2 cases). This is exactly the reason the TM sets were included in the benchmark suite: open-shell TM complexes are expected to constitute more of a challenge as compared to molecular systems made up of elements from the first three rows of the periodic table. It is also noted that the resetting approach can introduce a marginal increase in the iteration count, but this comes with the benefit that convergence is almost guaranteed. To summarize, in general, these results are clear empirical documentation of the benefits of the resetting procedure. More specifically, for systems with complicated open-shell electronic structures, the resetting mechanism can be the difference between frequent failures or not.

### Benchmarking of Three SCF Orbital Optimizers

**Figure 1:**
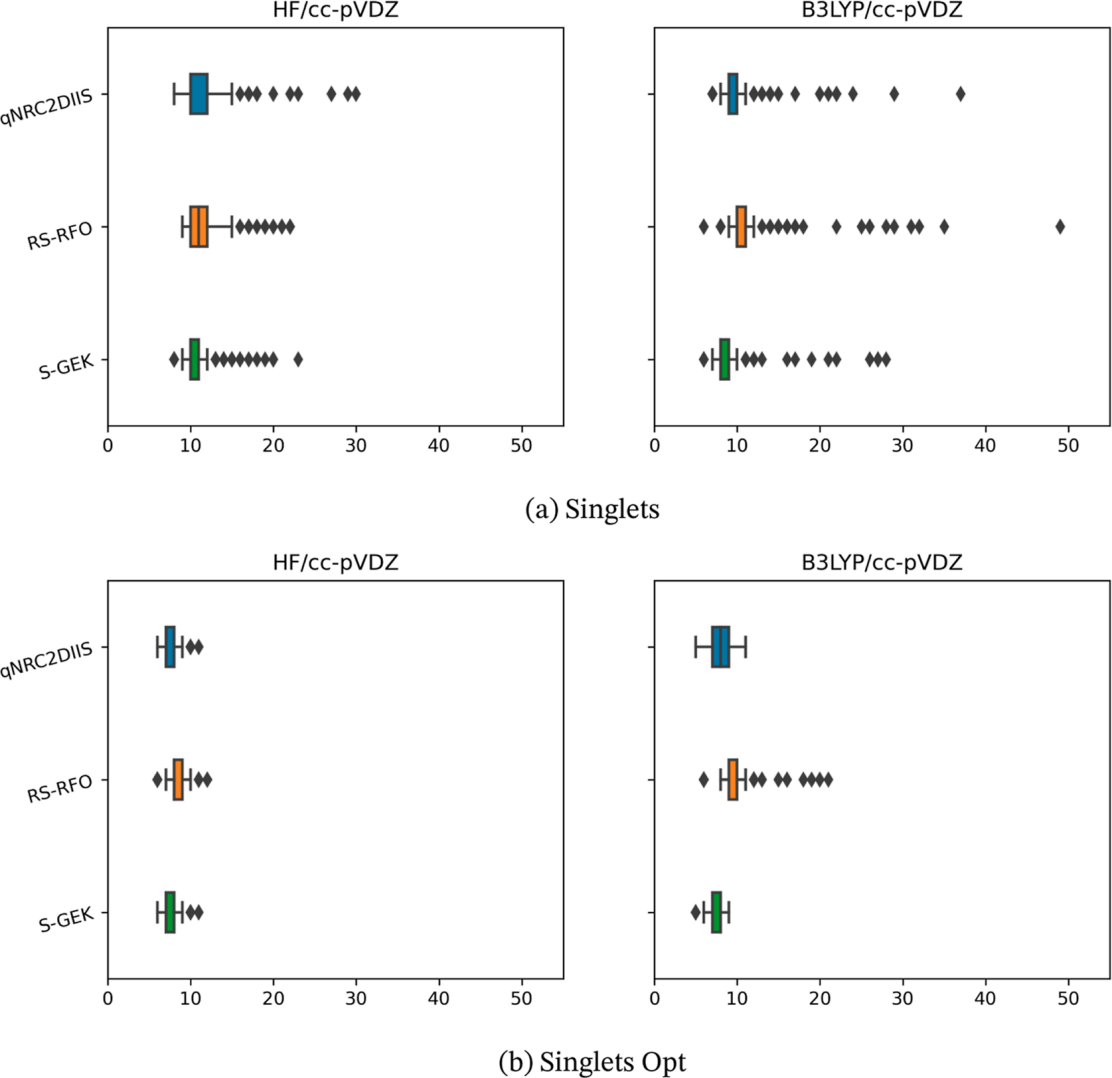
Benchmark iteration counts for the two benchmark sets of singlets—the singlets and singlets opt set are presented at the upper and the lower panels, a and b, respectively—evaluated at the HF and KS-DFT (B3LYP) level of theory using the r-GDIIS, the RS-RFO, and the S-GEK/RVO SCF/KS-DFT orbital optimization methods. The *x*-axis represents the iteration count until convergence. The data is presented with box-and-whisker plots, where the whisker range is derived by the 1.5 IQR-rule. Outliers are plotted as diamond-shaped signs.

**Figure 2:**
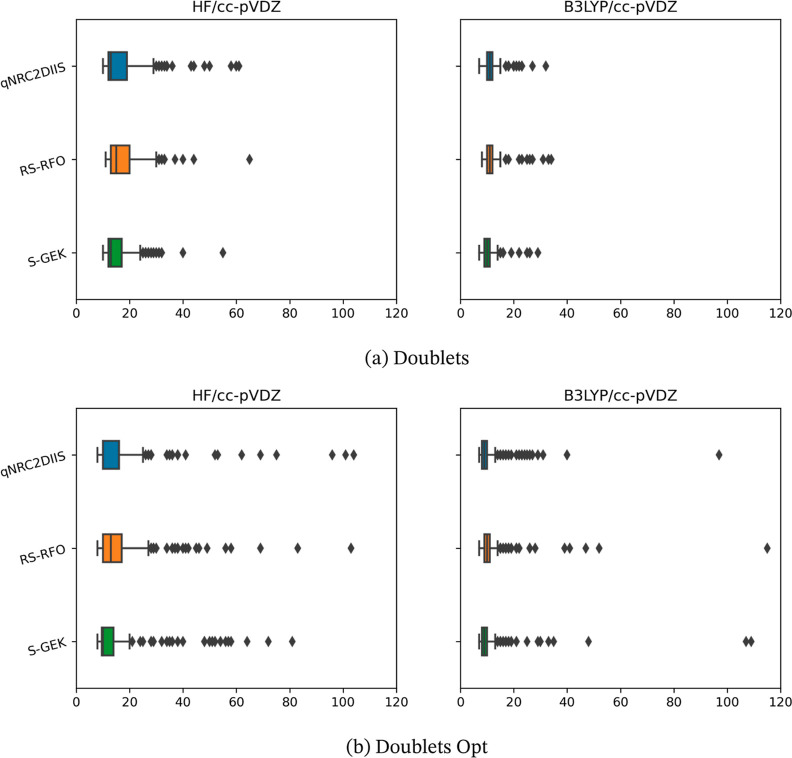
Benchmark iteration counts for the two benchmark sets of doublets—the doublets and doublets opt set are presented at the upper and the lower panels, a and b, respectively—evaluated at the HF and KS-DFT (B3LYP) level of theory using the r-GDIIS, the RS-RFO, and the S-GEK/RVO SCF orbital optimization methods. The *x*-axis represents the iteration count until convergence. The data is presented with box-and-whisker plots, where the whisker range is derived by the 1.5 IQR-rule. Outliers are plotted as diamond-shaped signs.

**Figure 3:**
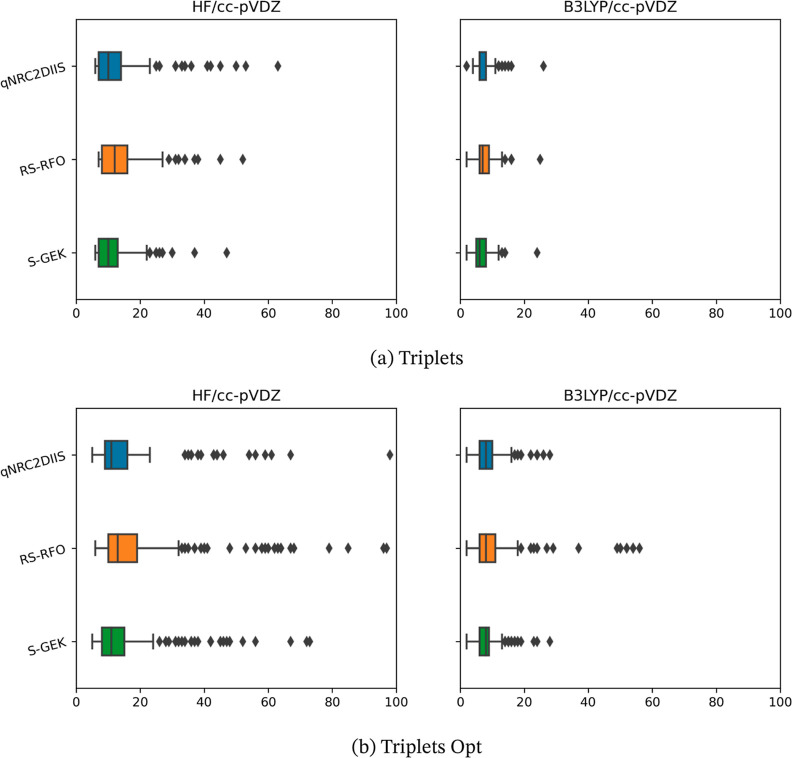
Benchmark iteration counts for the two benchmark sets of triplets—the triplets and triplets opt set are presented at the upper and the lower panels, a and b, respectively—evaluated at the HF and KS-DFT (B3LYP) level of theory using the r-GDIIS, the RS-RFO, and the S-GEK/RVO SCF/KS-DFT orbital optimization methods. The *x*-axis represents the iteration count until convergence. The data is presented with box-and-whisker plots, where the whisker range is derived by the 1.5 IQR-rule. Outliers are plotted as diamond-shaped signs.

**Figure 4:**
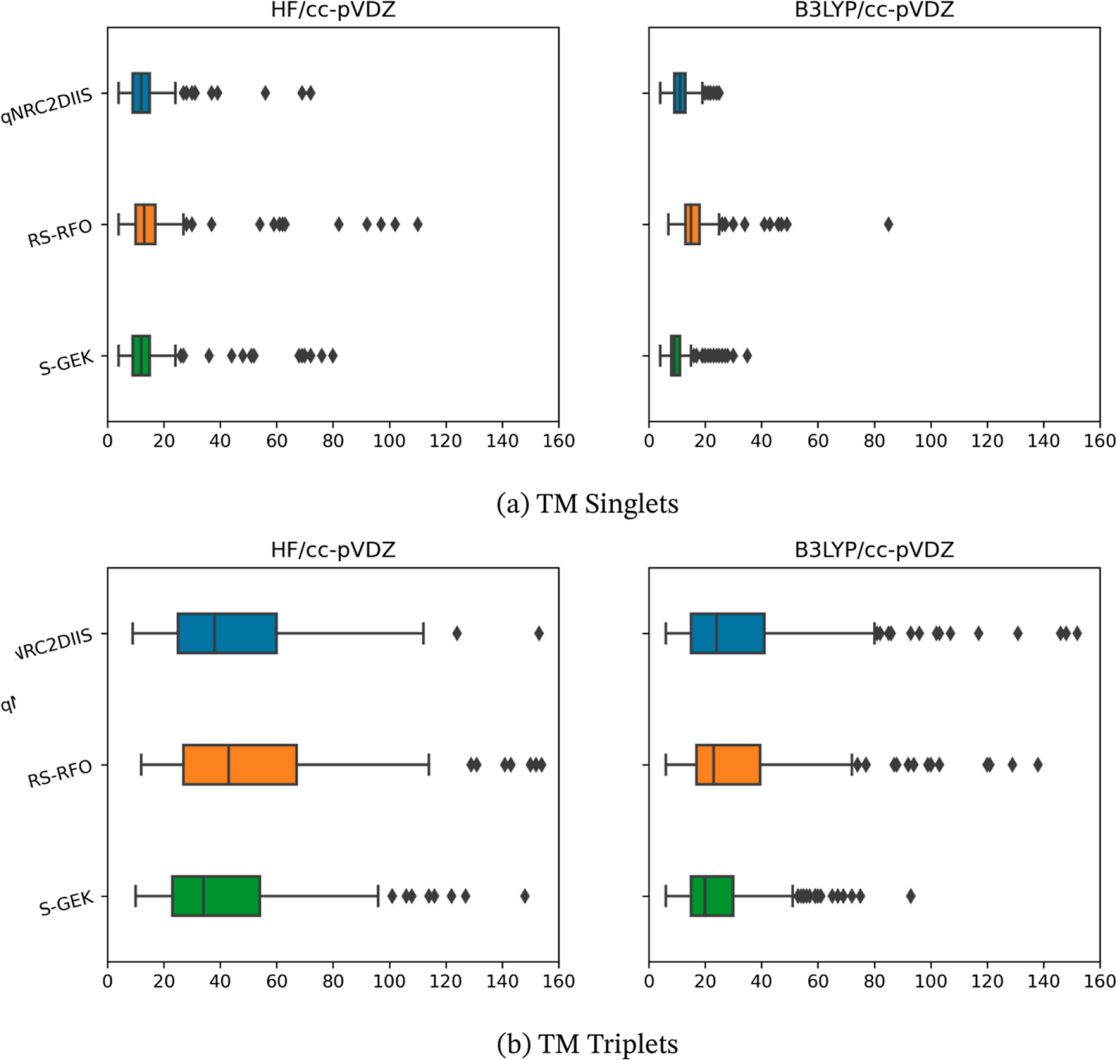
Benchmark iteration counts for the two benchmark sets of TMs—the TM singlets and TM triplets set are presented at the upper and the lower panels, a and b, respectively—evaluated at the HF and KS-DFT (B3LYP) level of theory using the r-GDIIS, the RS-RFO, and the S-GEK/RVO SCF/KS-DFT orbital optimization methods. The *x*-axis represents the iteration count until convergence. The data is presented with box-and-whisker plots, where the whisker range is derived by the 1.5 IQR-rule. Outliers are plotted as diamond-shaped signs.

The benchmark comparison among the r-GDIIS approach, the RS-RFO, and the S-RVO optimization procedures is now presented. First, Table 2 contains statistics with respect to average iterations within each benchmark set, the standard deviation of the same measure, the number of systems converging after more than 120 iterations, and the number of molecules for which the optimization did not converge before a total of 400 SCF iterations. Relying on a single statistical measure to evaluate performance is known to be problematic;^70^ in particular, the mean and standard deviation can be very sensitive to extreme values. Therefore, somewhat more detailed data are presented in Figures 1, 2, 3, and 4, in which the distribution of the iteration counts is represented by so-called box-and-whisker plots. Here, the box represents the first and third quartile, *Q*_1_ and *Q*_3_, respectively (so that it includes 50% of the cases); the line inside the box is the median value and second quartile. The whiskers extend to 1.5 times the interquartile range, IQR = *Q*_3_ – *Q*_1_, from the start and the end of the box but always ending on a data point within the range. In the presented results, the whiskers have different lengths since there are often no data points which are outside 1.5 times the IQR range to the left of the box, and the data before *Q*_1_ is rather compressed and is found close to the left of the *Q*_1_ marking. Any data points that are outside of the whiskers on either side are considered outliers and are represented explicitly. Note that the number of outliers is a function of how tight the distribution is. That is, a benchmark can have more outliers as a consequence of the underlying distribution being very tight. Hence, the number of outliers between the two benchmarks should be compared with care. The median and IQR values should be much less sensitive to extreme values than the mean and standard deviation.

**Table 2:** Benchmark Results for the Eight Sets of Benchmark Sets Compiled with the r-GDIIS Method, the RS-RFO, and the S-GEK/RVO Approaches^a^

	HF		
	r-GDIIS	RS-RFO	S-GEK/RVO
singlets	11.3(2.9)/0(0)	11.7(2.3)/0(0)	10.7(2.0)/0(0)
singlets opt	7.9(1.0)/0(0)	8.2(1.3)/0(0)	7.9(1.0)/0(0)
doublets	17.1(8.4)/0(0)	17.6(6.7)/0(0)	15.1(5.5)/0(0)
doublets opt	15.2(13.4)/0(0)	17.3(22.1)/1(0)	14.8(11.7)/0(0)
triplets	12.3(8.1)/0(0)	13.4(6.7)/0(0)	11.1(5.4)/0(0)
triplets opt	19.6(32.4)/8(0)	18.7(17.0)/0(0)	14.7(12.1)/0(0)
TM singlets	13.2(7.6)/0(0)	15.7(13.6)/0(0)	13.8(10.7)/0(0)
TM triplets	47.8(36.9)/7(2)	49.5(27.2)/7(0)	41.8(24.3)/4(0)

	B3LYP		
	r-GDIIS	RS-RFO	S-GEK/RVO
singlets	9.8(3.1)/0(0)	11.2(4.4)/0(0)	9.0(3.0)/0(0)
singlets opt	8.1(1.4)/0(0)	9.7(2.0)/0(0)	7.5(1.1)/0(0)
doublets	11.1(3.0)/0(0)	11.8(3.5)/0(0)	10.5(2.9)/0(0)
doublets opt	11.4(12.2)/1(0)	12.4(14.9)/1(0)	10.7(10.1)/0(0)
triplets	7.0(2.5)/0(0)	7.4(2.8)/0(0)	6.7(2.3)/0(0)
triplets opt	8.6(4.1)/0(0)	9.8(7.9)/0(0)	8.1(3.8)/0(0)
TM singlets	11.4(3.8)/0(5)	16.5(7.4)/0(2)	10.0(4.3)/0(0)
TM triplets	33.5(29.6)/7(2)	31.4(22.9)/3(0)	24.5(14.5)/0(0)

a The reported numbers are the average number of iterations post SCF startup (the standard deviation)/the number of molecules for which more than 120 total iterations were needed (the number of molecules which did not converge before a total of 400 SCF iterations).

For all cases, upon monitoring both the average iterations and the standard deviations, one can observe the following trends. The KS-DFT optimizations convergence is typically faster compared to HF. Moreover, the convergence tends to be swifter for the benchmark sets that represent molecules at equilibrium structures as compared to that of molecules at nonequilibrium structures—i.e., within the singlet and doublet sets. This is, in particular, true for the S-RVO optimizer. Exceptions exist; for example, the transition metal HF singlet optimizations in general converge faster than the corresponding KS-DFT optimizations. The most striking result is, however, that for all eight test sets, using HF or KS-DFT, the S-RVO procedure shows superior performance as compared to that of both the r-GDIIS and the RS-RFO procedures, with the possible exception of the TM singlets with HF. For the test sets of the organic molecules, this advantage is modest but consistent. For the TMs, the S-RVO demonstrates a significant advantage as compared to the two other optimization methods, this in particular for the open-shell (triplets) benchmark test suite; the average iterations are reduced from 47.8 to 41.8 when comparing r-GDIIS and S-GEK/RVO. For the KS-DFT optimization, the corresponding results are reduced from 33.5 to 24.5. The table and the corresponding figures also readily show that the observed dispersion is significantly reduced for the S-RVO method compared to that for the two other methods, and the extreme values tend to be lower. It is noted that in some cases, there are a handful of outliers beyond the maximum value displayed in the figures but never for the S-RVO method. Finally, one can observe that the S-RVO procedure is the only one for which there is no single case in which the optimization did not converge. It is evident that the nonparametric surrogate model, although just expressed in a subspace, is a significant step forward toward a swift and robust SCF/KS-DFT orbital optimization method.

Briefly, on comparison of the r-GDIIS versus the RS-RFO method, the results are a bit disappointing. The RS-RFO approach, as implemented in this study, does not exhibit any superiority over the r-GDIIS; a comparison with the GDIIS would of course leave a completely different verdict. The only cases where there seems to be some advantage of the RS-RFO as compared to the r-GDIIS approach can be found in the statistics of the KS-DFT optimizations of the TMs. The origin of the generally slower convergence rate can be only speculated about. Is it because the step restriction is too tight? Considering that both methods are molded in the frame of a second-order method (both are quasi-Newton methods in some respect), one would not expect a significant difference in performance. Hence, the thresholds used for activating the step restriction and the underlying ad-hoc procedure involved in these decisions can be suspected. This will not, however, be analyzed any further here, especially when the restricted variance approach of the RVO procedure demonstrates a significant advantage over the step restriction procedure of the RS-RFO method; therefore, the RVO procedure based on S-GEK should be the norm.

**Figure 5:**
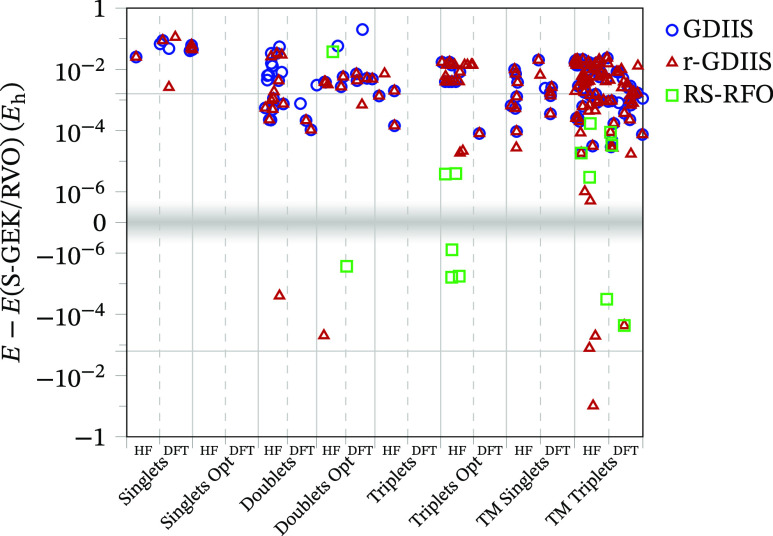
Difference between converged energies with respect to the S-GEK/RVO result. Only differences larger than 5 × 10^–7^*E*_h_ in absolute value are represented. Note that the *y* axis is divided into positive and negative sections, each of them in logarithmic scale. The horizontal grid lines mark ±1 kcal/mol.

In addition to the iteration count, it is also interesting to analyze the quality of the converged wave function or orbitals. We do this by comparing the final energies obtained in the calculations, taking the S-GEK/RVO results as a reference since it was the method that converged in all cases. Most of the 4160 total structures, considering separately the HF and B3LYP calculations, resulted in the same converged energy (when converged) with the four methods: GDIIS, r-GDIIS, RS-RFO, and S-GEK/RVO. Only in 199 cases is the energy difference larger than 5 × 10^–7^*E*_h_. These are represented in Figure 5, where it is evident that most differences are positive, i.e., the S-GEK/RVO method converges to a lower (when not equal) energy than the other methods, except in a dozen of cases. It is also clear that GDIIS and r-GDIIS tend to differ more from S-GEK/RVO than from RS-RFO. Moreover, it can be observed that most differences are found in the sets of TM complexes (123 cases) and, in general, more in HF than in B3LYP calculations (144 vs 55).

This section ends with some timing observations and suggestions for future developments. First, it was noted that the RS-RFO approach has significantly longer timings per iteration as compared to that of the r-GDIIS approach. This most likely has its origin in that the former handles the full parameter space, while the r-GDIIS only works in a very limited subspace. Second, the S-GEK/RVO was adopted after the RS-RFO procedure, and in this context, the S-GEK/RVO did not add significant additional timing to the underlying RS-RFO method. Considering the consistently better performance of the r-GDIIS as compared to that of RS-RFO—both for the convergence rate and the timings, one should explore the combination of the r-GDIIS and the S-GEK/RVO procedure for even better and faster convergence.

## Conclusions

In this report, three SCF/KS-DFT orbital optimization schemes have been investigated using a large benchmark test suite to evaluate performance characteristics. First, a resetting version of the GDIIS procedure, the r-GDIIS approach, was presented. This approach uses a number of different criteria to decide when the DIIS depth needs to be reset in order to avoid divergence or a poor convergence rate. Second, an RS-RFO scheme was presented. This method is facilitated by the use of the Davidson procedure in connection with an on-the-fly Hessian update procedure as the rational function equations are solved. Finally, a novel implementation of the GEK adapted to a subspace formalism, S-GEK, was presented and implemented in the context of an RVO procedure. The benchmark calculations included in total an excess of 2000 cases, singlet, doublet, and triplet state organic molecular systems, and singlet and triplet state TM complexes, about half of the structures corresponding to equilibrium molecular structures. The results of the benchmarking gave a clear indication that S-GEK is superior to any of the conventional state-of-the-art methods of SCF/KS-DFT orbital optimization explored in this study. In particular, it was demonstrated that for TMs, the improvements in convergence rates are impressive and robust. Moreover, the new method exhibits sturdy characteristics with respect to handling difficult cases; in all, there was not a single instance in which the S-GEK/RVO optimization failed to complete the optimization procedure.

To conclude, the paper suggests that the S-GEK/RVO procedure should be explored in conjunction with the r-GDIIS approach for optimal CPU timings. In addition, considering that the S-GEK/RVO approach is a nonparametric surrogate model, there is no obvious requirement that it needs to start in the so-called quadratic region of the parameter space. That is, it should be worthwhile to investigate to what extent the iterations in the SCF startup scheme can be reduced by switching over to the S-GEK/RVO approximation earlier in the optimization procedure.

## Appendix

The SCF optimization methods described, implemented and tested in this work are for the most part based on a second-order Taylor expansion, and therefore, they work best when the wave function is relatively close to a minimum, such that the energy is approximately quadratic with respect to **κ**. Over the years, experience has shown that far from a minimum, performance tends to be poor, and convergence can be an issue. To address this, several “SCF acceleration” schemes have been proposed, whose aim is to guide the optimization in a fast and robust manner toward a quadratic region where efficient optimization methods can perform best. The process from the beginning of a calculation to the point where the final optimization method (GDIIS, r-GDIIS, RS-RFO, or S-GEK/RVO) is activated is what is referred to in this work as “SCF startup” and includes both the definition of the starting orbitals and the initial SCF iterations. Given that this process is identical regardless of the chosen final method, the iterations used for it are excluded from the iteration counts reported in the main text. Although the startup process has not been the subject of this work, it will be briefly described in this Appendix for completeness. It is the default startup in OpenMolcas,^59^ except where noted.

### Starting Orbitals

The SCF procedure must begin with a starting set of molecular orbitals that define the reference Slater determinant. While in principle any orthonormal set of orbitals could be used, the process will benefit from an initial guess as close as possible to the final solution, facilitating rapid convergence to the ground state and avoiding spurious convergence to unphysical solutions. The performance of any optimization method is therefore dependent on how the starting orbitals are defined. In all cases, the default in OpenMolcas^59^ have been used. Nevertheless, since the process by which these starting orbitals are defined has not, to our knowledge, been previously described, we take the opportunity of doing so in this Appendix, noting again that this is not strictly part of this work.

The initial (guess) orbitals for the SCF procedure are obtained by diagonalization of a synthetic model Fock matrix ***F***^m^. This model Fock matrix is constructed in AO basis, as a sum of atomic blocks, with interatomic blocks set to 0, i.e.,

35

where *A* and *B* refer to atoms or centers for basis functions. Each atomic block corresponds to a model Fock operator that is diagonal in a set of orthonormal reference functions

36

where η are the energies of the reference functions. The reference atomic Fock matrix is transformed to an arbitrary basis set via the overlaps between the basis set and the reference functions (***S***^*A*,ref^, a rectangular matrix) and among the basis set functions (***S***^*A*^, a symmetric matrix)

37

38

39

Once *F*^m^ is obtained by eq 35, it is first transformed to an orthonormal basis, defined by canonical orthonormalization. If ***S*** is the overlap matrix of the full system in the desired basis, the matrix ***L*** is defined as

40

where ***V*** is the matrix of eigenvectors of ***S*** and **σ** is the diagonal matrix of its eigenvalues. Then, the transformed ***F***^m^ is

41

Note that, in the event of a monoatomic system, the factors  and ***S*** in eqs 39 and 41 will cancel out, but in general ***S*** ≠ ***S***^*A*^. Diagonalization of  yields a set of eigenvectors and eigenvalues that can be used as starting orbital coefficients and energies for the SCF procedure.

The reference functions from which the atomic Fock matrices, eq 36, are constructed are quite limited; only valence functions are typically included. Any reasonable calculation will contain many more functions that thus span a larger Hilbert space. As a consequence, the diagonalization of  produces a large number of orbitals with zero or very small energies, which are not resolved. In order to generate a more useful and well-defined set of orbitals for this “virtual” space, they are subject to a further diagonalization using the kinetic energy operator.

The subset of eigenvectors of  corresponding to eigenvalues larger than −10^–3^*E*_h_, ***C***_virt_, is used to obtain the kinetic energy matrix over the virtual space, from the full kinetic energy matrix in AO basis

42

and they are transformed to diagonalize the ***T***_virt_

43

where ***U*** are the eigenvectors of ***T***_virt_. The energies of these orbitals are taken as the corresponding eigenvalues, shifted by 3.0 *E*_h_, to ensure that they are well above the rest.

The reference functions and η values used in eqs 36 and 39 are the lowest-lying atomic natural orbitals and their energies, as defined by the ANO-RCC basis set.^71−73^ The energies are defined in the files distributed with the OpenMolcas source code,^59^ for convenience, the values relevant for the calculations in this work are given in the Supporting Information.

### Initial Optimization Procedures

Once the starting orbitals are defined, an initial wave function and density matrix are set up by occupying the lowest-lying orbitals, according to the aufbau principle. Alternatively, an initial “Fermi aufbau” procedure is employed, in which the orbitals are assigned fractional occupations consistent with a temperature *T*_F_ and Fermi energy *E*_F_

44

where *n*_max_ is 2 for restricted SCF and 1 for unrestricted SCF calculations, ε_*i*_ are the orbital energies, and *E*_F_ is defined in such a way that the sum of *n*_*i*_ matches the total number of electrons in the system, i.e., the only input parameter is *T*_F_. The default initial value for *T*_F_ is 0.5 *E*_h_, and it is multiplied by 0.46 each iteration. When *T*_F_ reaches 0.01 *E*_h_ or lower and the energy change from the previous iteration is below 0.01 *E*_h_, the aufbau procedure is terminated, and orbitals are assigned only 0 or *n*_max_ occupations. This Fermi aufbau process is enabled automatically whenever the starting orbital energies are not considered reliable enough for a straightforward aufbau occupation. In the calculations presented in this work, this happens only for the triplets (in the triplets, triplets opt, and TM triplets sets) and for the anionic singlets (cases 000–095 in the TM singlets set).

The optimization method applied during the initial iterations is the “energy-DIIS” (EDIIS),^8^ which, unlike the methods described in the Theory section, is not formulated in terms of orbital rotations but as a more straightforward SCF approach with successive construction and diagonalization of a Fock matrix.^74^ In EDIIS, the Fock matrix that is diagonalized at a given iteration is not the one generated by the “current” density matrix ***D***_*m*_, but by an interpolated density matrix

45

The *c*_*i*_ coefficients are obtained by minimizing the energy function

46

where ***F***_*i*_ are the Fock matrices corresponding to the density matrices. When  is found, it is used for building an interpolated Fock matrix , and diagonalizing this yields a new set of orbitals used in the next iteration. The EDIIS optimization proceeds until the energy change is below 0.1 *E*_h_, and the maximum absolute value of the off-diagonal elements of  is smaller than 0.15.

After the EDIIS method has converged, the optimization switches to C^2^-DIIS,^42^ in which the coefficients are first constrained to ∑_1_^*m*^*c*_*i*_^2^ = 1 instead of ∑_1_^*m*^*c*_*i*_ = 1 (and negative coefficients are allowed, so extrapolations are possible). In contrast to the GDIIS approach described in the Theory section, no quasi-Newton–Raphson-like step is performed after. This method continues until the off-diagonal elements of the density matrix are below 0.075, in absolute value. At that point, the final optimization method (GDIIS, r-GDIIS, RS-RFO, or S-GEK/RVO in this work) is enabled.

## Supporting Information Available

Model energy values used for the definition of the initial orbitals (PDF)
Coordinates for all molecular systems computed in this work, converged energy and iteration count for each calculation (ZIP)
