# Supplementary material for: A Story of Three Levels of Sophistication in SCF/KS-DFT Orbital Optimization Procedures
Source: J Phys Chem A. 2024 Mar 14;128(12):2472–86. doi: 10.1021/acs.jpca.3c07647 (PMC10983011; doi:10.1021/acs.jpca.3c07647)
Supplement: Supplementary file 1 — jp3c07647_si_001.pdf [file jp3c07647_si_001.pdf]

Supporting Information: A Story of Three Levels of Sophistication in SCF/KS-DFT Orbital Optimization Procedures

Daniel Sethio,[†,‡] Emily Azzopardi,[†] Ignacio Fdez. Galván,[*,†] and Roland Lindh[*,†,¶]

†*Department of Chemistry – BMC, Uppsala University, P. O. Box 576, SE-75123 Uppsala, Sweden*

‡*Department of Chemistry – Ångström, Uppsala University, P. O. Box 538, SE-75121 Uppsala, Sweden*

¶*Uppsala Center for Computational Chemistry (UC_3), Uppsala University, PO Box 576, SE-751 23 Uppsala. Sweden*

E-mail: Ignacio.Fernandez@kemi.uu.se; roland.lindh@kemi.uu.se

Contents of the `data.zip` file:

- **Coords**
  - `Doublets`: Organic molecules with odd number of electrons
  - `Doublets_Opt`: Organic molecules with odd number of electrons, geometry optimized as a doublet
  - `Singlets_Triplets`: Organic molecules with even number of electrons, used with both singlet and triplet multiplicity

- Singlets_Triplets_Opt: Organic molecules with even number of electrons, geometry optimized as a singlet, used with both singlet and triplet multiplicity
- TM_Singlets_Triplets: Molecules containing transition metal atoms, with even number of electrons (total charge indicated in comment line), used with both singlet and triplet multiplicity

- **Iterations**

  Number of iterations (post SCF startup) until convergence, negative numbers indicate no convergence in 400 iterations. One csv file for each set of molecular systems. In each file, one column for each method. Rows ordered according to the numerical order of the structures in the Coords directory (from 000 to 234, 264 or 274).

- **Energies**

  Final SCF/DFT energy for converged calculations, 0.0 indicates no convergence in 400 iterations. One csv file for each set of molecular systems. In each file, one column for each method. Rows ordered according to the numerical order of the structures in the Coords directory (from 000 to 234, 264 or 274).

Table S1: Energies (η values), in E_h, for the lowest-lying functions of the ANO-RCC basis set, classified by angular moment type.

Atom	n	s	p	d	f
H	1	−0.5000			
B	1	−7.6963			
	2	−0.3109	−0.4945		
C	1	−11.3276			
	2	−0.7053	−0.4354		
N	1	−15.6340			
	2	−0.9454	−0.5707		
O	1	−20.6840			
	2	−1.2475	−0.6362		
F	1	−26.4081			
	2	−0.7312	−1.5764		

Table S1: (Continued) Energies (η values), in E_h, for the lowest-lying functions of the ANO-RCC basis set.

Atom	n	s	p	d	f
Si	1	−68.9761			
	2	−6.1823	−4.2544		
	3	−0.5416	−0.2969		
P	1	−80.1893			
	2	−7.5472	−5.3996		
	3	−0.6992	−0.3916		
S	1	−92.2944			
	2	−9.0544	−6.6828		
	3	−0.8845	−0.4384		
Cl	1	−105.1992			
	2	−10.6632	−8.0478		
	3	−1.0865	−0.5114		
Sc	1	−166.8194			
	2	−19.2606	−15.6810		
	3	−2.5908	−1.5712	−0.3376	
	4	−0.2116			
Ti	1	−184.3952			
	2	−21.6486	−17.8164		
	3	−2.9047	−1.7968	−0.4318	
	4	−0.2226			
V	1	−202.8621			
	2	−24.4289	−20.0609		
	3	−3.2247	−2.0240	−0.5004	
	4	−0.2334			
Cr	1	−222.2238			
	2	−26.7836	−22.4137		
	3	−3.5526	−2.2531	−0.5609	
	4	−0.2438			
Mn	1	−242.4774			
	2	−29.5268	−24.8831		
	3	−3.8813	−2.4902	−0.6281	
	4	−0.2527			
Fe	1	−263.6739			
	2	−32.4391	−27.5017		
	3	−4.2511	−2.7542	−0.6378	
	4	−0.2636			

Table S1: (Continued) Energies (η values), in E_h, for the lowest-lying functions of the ANO-RCC basis set.

Atom	n	s	p	d	f
Co	1	−285.7690			
	2	−35.4818	−30.2392		
	3	−4.6328	−3.0259	−0.6522	
	4	−0.2745			
As	1	−438.8931			
	2	−57.7704	−50.4868		
	3	−8.2735	−5.9334	−2.0674	
	4	−0.7031	−0.3690		
Se	1	−468.0186			
	2	−62.3329	−54.6552		
	3	−9.2130	−6.7256	−2.5908	
	4	−0.8615	−0.4047		
Br	1	−498.1383			
	2	−67.0881	−58.9949		
	3	−10.1941	−7.5441	−3.1588	
	4	−1.0223	−0.4578		
Zr	1	−664.8981			
	2	−94.7816	−84.3327		
	3	−16.6848	−13.1727	−7.3888	
	4	−2.5062	−1.4960	−0.3180	
	5	−0.2141			
Nb	1	−701.3320			
	2	−100.9107	−89.9254		
	3	−18.0996	−14.3967	−8.3333	
	4	−2.7731	−1.6876	−0.3869	
	5	−0.2259			
Mo	1	−738.8075			
	2	−107.2399	−95.6949		
	3	−19.5538	−15.6589	−9.2934	
	4	−3.0371	−1.8794	−0.4484	
	5	−0.2364			
Tc	1	−777.3318			
	2	−113.7715	−101.6377		
	3	−21.0500	−16.9461	−10.2905	
	4	−3.3032	−2.0686	−0.5212	
	5	−0.2447			

Table S1: (Continued) Energies (η values), in E_{h}, for the lowest-lying functions of the ANO-RCC basis set.

Atom	n	s	p	d	f
Ru	1	−816.9370			
	2	−120.5324	−107.7870		
	3	−22.6111	−18.3083	−11.3407	
	4	−3.5868	−2.2722	−0.5438	
	5	−0.2539			
Rh	1	−857.4466			
	2	−127.3313	−113.9397		
	3	−24.0465	−19.5146	−12.2635	
	4	−3.7139	−2.3417	−0.4305	
	5	−0.2387			
I	1	−1224.1148			
	2	−192.8325	−173.0175		
	3	−40.4922	−33.8858	−23.9316	
	4	−7.7545	−5.5803	−2.3087	
	5	−0.8775	−0.4040		
Hf	1	−2409.6123			
	2	−417.3600	−367.7121		
	3	−97.2850	−82.0769	−63.4090	
	4	−20.7111	−15.6009	−8.7448	−1.1375
	5	−2.9393	−1.6044	−0.2688	
	6	−0.2368			
Ta	1	−2485.8453			
	2	−432.4668	−380.3718		
	3	−101.3093	−85.4254	−66.1645	
	4	−21.8035	−16.4671	−9.3707	−1.4748
	5	−3.1866	−1.7721	−0.3273	
	6	−0.2505			
W	1	−2563.6007			
	2	−447.9470	−393.2860		
	3	−105.4530	−88.8647	−68.9882	
	4	−22.9376	−17.3685	−10.0130	−1.8313
	5	−3.4387	−1.9467	−0.3817	
	6	−0.2641			
Re	1	−2642.8979			
	2	−463.8023	−406.4495		
	3	−109.7119	−92.3834	−71.8796	
	4	−24.1076	−18.2874	−10.6789	−2.2067
	5	−3.6920	−2.0893	−0.4421	
	6	−0.2744			

Table S1: (Continued) Energies (η values), in E_{h}, for the lowest-lying functions of the ANO-RCC basis set.

Atom	n	s	p	d	f
Os	1	−2723.7818			
	2	−480.0615	−419.8863		
	3	−114.1080	−96.0057	−74.8565	
	4	−25.3353	−19.2520	−11.3843	−2.6191
	5	−3.9674	−2.2942	−0.4604	
	6	−0.2869			
Ir	1	−2806.1428			
	2	−495.5910	−433.4585		
	3	−118.5024	−99.5964	−77.7787	
	4	−26.4766	−20.1289	−11.9850	−2.9200
	5	−4.1242	−2.3805	−0.3820	
	6	−0.2917			
